# Dual-Site Inflammation During Adalimumab Therapy: Birdshot Chorioretinopathy Complicated by Paradoxical Psoriasis

**DOI:** 10.7759/cureus.107540

**Published:** 2026-04-22

**Authors:** Diana Naumkinaite, Melita Virpsaite, Ieva Radaviciute, Tadas Raudonis, Andrius Cimbalas

**Affiliations:** 1 Faculty of Medicine, Vilnius University, Vilnius, LTU; 2 Clinic of Infectious Diseases and Dermatovenereology, Faculty of Medicine, Vilnius Univeristy, Vilnius, LTU; 3 Clinic of Ear, Nose, Throat and Eye Diseases, Faculty of Medicine, Vilnius University, Vilnius, LTU

**Keywords:** adalimumab (humira), birdshot chorioretinopathy, cystoid macular edema, paradoxical psoriasis, posterior uveitis

## Abstract

Birdshot chorioretinopathy is a chronic posterior uveitis that often requires systemic immunomodulatory therapy, yet biologic agents, particularly tumor necrosis factor alpha (TNF-α) inhibitors, may trigger paradoxical cutaneous inflammation and complicate multidisciplinary management.

A 32-year-old man was evaluated for progressive right eye (RE) visual decline and long-standing floaters. Examination showed bilateral posterior uveitis with chorioretinitis and retinal vasculitis, more severe in the RE, with significant cystoid macular edema (CME) and leakage on fluorescein angiography (FA). Infectious and systemic causes were excluded, and human leukocyte antigen A29 (HLA-A29) was not detected. Systemic methylprednisolone induced partial anatomic improvement but was limited by recurrence during tapering and by intraocular pressure (IOP) elevation, prompting escalation with mycophenolate mofetil and later adalimumab therapy for persistent, refractory CME. After an insufficient response, an intravitreal dexamethasone implant achieved edema regression but was followed by ocular hypertension requiring sustained IOP-lowering therapy. During the same treatment course, new-onset palmoplantar pustular and scalp lesions developed, with steroid-dependent fluctuations, and histopathology showed mild spongiotic dermatitis with Langerhans cell microabscesses; the overall presentation was considered consistent with adalimumab-associated paradoxical psoriasis. Cutaneous disease showed only transient benefit from topical therapy and phototherapy, and an interleukin-17 (IL-17) inhibitor was recommended but declined by the patient.

This case illustrates how anti-TNF-α therapy may be insufficient for ocular inflammatory control while precipitating paradoxical psoriasis. Optimal outcomes rely on coordinated ophthalmology-dermatovenereology care when ocular disease activity and treatment-emergent skin complications evolve in parallel.

## Introduction

Birdshot chorioretinopathy is a rare, chronic, and predominantly bilateral entity within the spectrum of posterior uveitis, defined by inflammatory involvement of the choroid and retina and frequently accompanied by retinal venous vasculitis [1]. Although the condition is strongly associated with the human leukocyte antigen A29 (HLA-A29) allele, HLA-A29-negative birdshot-like phenotypes have been reported, indicating that the genetic marker alone is insufficient to confirm or exclude the diagnosis. In this setting, the differential diagnosis should be broadened to include alternative causes of birdshot-like posterior uveitis, with targeted evaluation for immunodeficiency or conditions mimicking uveitis, as such presentations may represent broader systemic involvement [2]. Moreover, tumor necrosis factor alpha (TNF-α) inhibitors, despite an established role in inflammatory control, may induce paradoxical immune-mediated reactions, thereby complicating both treatment selection and long-term management [3]. This case report presents an HLA-A29-negative birdshot-like posterior uveitis with refractory cystoid macular edema (CME), in which management was complicated by paradoxical psoriasis during adalimumab therapy and by limited corticosteroid suitability due to ocular hypertension.

## Case presentation

A 32-year-old man presented in May 2024 for a scheduled ophthalmology consultation at the Department of Ophthalmology, Vilnius University Hospital Santaros Klinikos, Vilnius, Lithuania, because of progressively worsening vision in the right eye (RE) and long-standing floaters in both eyes. According to the patient, floaters and visual acuity in the right eye had been reduced since childhood, and he reported a more pronounced deterioration since 2023. Prior to presentation, the patient had recently started topical dorzolamide for intraocular pressure (IOP) reduction.

Ophthalmic evaluation revealed bilateral posterior uveitis with chorioretinitis in both eyes, more prominent in RE than in the left eye (LE). Best-corrected visual acuity was 0.08 in the RE and 1.25 in the LE, with 17 mmHg IOP and no clinically significant anterior segment abnormalities. Vitreous inflammatory cells were graded up to 2+ in the RE and 1+ in the LE. Fundus examination showed optic discs with sharp margins and normal in color, with preservation of the foveolar reflex. Mild perivascular sheathing was noted in the parapapillary region. In RE, depigmented lesions were present in the inferior peripheral retina, and multiple scattered hypopigmented spots were observed in the mid-periphery, resembling birdshot lesions (small shotgun pellets).

**Figure 1:**
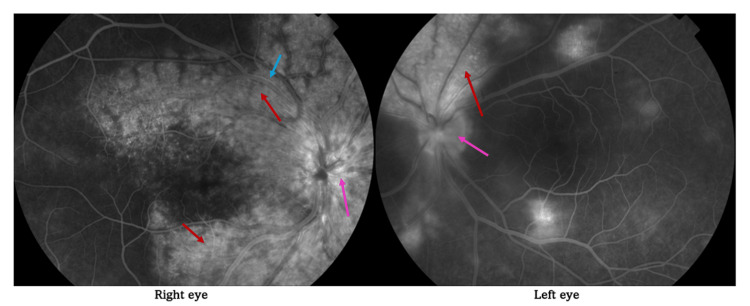
Fluorescein angiography findings Arrows indicate parapapillary (pink) and perivascular (red) punctate confluent hyperfluorescence (dye leakage along the vascular arcades). Blue arrow indicates vascular wall hyperfluorescence.

Optical coherence tomography (OCT) confirmed marked CME in the RE and an epiretinal membrane in the LE. Fluorescein angiography (FA) demonstrated parapapillary and perivascular (along the vascular arcades) punctate confluent hyperfluorescence with late-phase dye leakage, as well as vascular wall hyperfluorescence consistent with retinal vasculitis (Figure 1).

The clinical presentation was consistent with birdshot chorioretinopathy; therefore, systemic therapy with oral methylprednisolone was initiated at 48 mg/day for two weeks, followed by a gradual tapering to 32 mg/day at the one-month follow-up, while topical nepafenac was added for the RE. Before this consultation, the diagnostic work-up had already been initiated by another specialist, and the relevant tests had been ordered: complete blood count, C-reactive protein, erythrocyte sedimentation rate, and rheumatoid factor were within normal ranges. Infectious causes (tuberculosis, toxoplasmosis, human immunodeficiency virus infection, syphilis, and borreliosis) and systemic etiologies (sarcoidosis) were excluded, and HLA-A29 was not detected.

In June 2024, while continuing methylprednisolone at 32 mg/day, OCT demonstrated a reduction of CME in the RE, yet early atrophic changes of the photoreceptor outer segment layer became apparent. IOP increased in both eyes to approximately 25 mmHg, prompting the initiation of dorzolamide and the continuation of tapering systemic glucocorticosteroids (GCS). In August 2024, after the patient discontinued topical therapy and reached a methylprednisolone dose of 6 mg/day, OCT documented a recurrence of CME in the RE with a considerable increase in central retinal thickness. Treatment was intensified by re-escalating the systemic GCS dose and adding mycophenolate mofetil as an immunosuppressive agent.

Despite escalation, CME in the RE persisted and progressed between October and December 2024, without meaningful improvement in visual function. Brain magnetic resonance imaging did not support a demyelinating process; therefore, biologic therapy with adalimumab (Humira) was initiated in December 2024, concomitantly completing methylprednisolone tapering according to protocol. After approximately two months of adalimumab therapy, CME in the RE remained severe (central retinal thickness up to 1090 µm), and an intravitreal dexamethasone implant (Ozurdex, AbbVie, North Chicago, IL, USA) was administered in the RE in February 2025. Pre-procedural OCT also revealed abundant intraretinal fluid in the macula of the RE.

**Figure 2:**
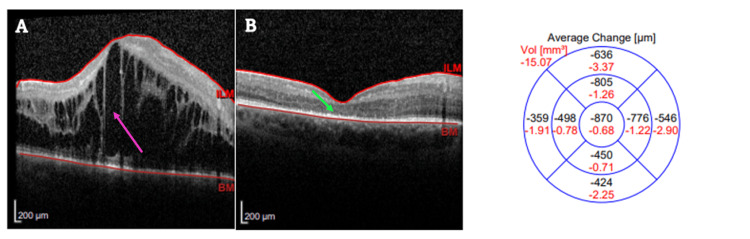
Optical coherence tomography of the right eye before and after the first intravitreal dexamethasone implant (A) Baseline optical coherence tomography of the right eye showing cystoid macular edema with multiple intraretinal cystic spaces (pink arrow); (B) follow-up optical coherence tomography two months after intravitreal dexamethasone implant; the green arrow indicates central photoreceptor atrophy. The thickness change map shows a central retinal thickness decrease of 870 µm. Abbreviations: ILM = internal limiting membrane, BM = Bruch’s membrane, Vol = volume

During the follow-up in April 2025, OCT confirmed regression of CME in RE (Figure 2), whereas central photoreceptor atrophy became evident. In addition, IOP in the RE acutely increased to 46.9 mmHg, accompanied by a new complaint of foggy vision. Topical dorzolamide/timolol 20 mg/5 mg/mL (twice daily) and systemic acetazolamide (750 mg once daily for four days, followed by 500 mg once daily for one week) were initiated, with subsequent addition of topical brimonidine and discontinuation of acetazolamide because of intolerance (dyspepsia and paresthesias).

In the spring of 2025, while receiving treatment for birdshot chorioretinopathy and trying to control IOP, the patient developed palmoplantar pustular eruptions and scalp involvement for the first time in his life. The course of the cutaneous disease was dependent on systemic GCS, improving during treatment and relapsing with dose reduction or discontinuation. After GCS withdrawal, dermatovenereology assessment documented erythematous scaly plaques on the hands and feet, several pustules on the soles, and serous-fluid-filled vesicles on the palms, with digital dermoscopy demonstrating erythema, pustules, and vesicles. On the scalp, infiltrated erythematous plaques covered with white scale were observed; the Auspitz sign was positive, and digital dermoscopy revealed dotted capillaries, white scale, and erythema. A punch skin biopsy showed mild spongiotic dermatitis/eczema with scattered Langerhans cell microabscesses. Based on the clinical presentation, steroid-dependent course, and histopathological findings, the condition was considered consistent with paradoxical psoriasis induced by the TNF-α inhibitor adalimumab; consequently, it was discontinued in June 2025. Dermatologic therapy was initiated with topical agents (emollients containing 10% urea; clobetasol propionate applied over the emollient; and mometasone furoate for the scalp) and phototherapy (psoralen + ultraviolet A for the palms and soles and narrowband ultraviolet B 311 nm for the scalp).

**Figure 3:**
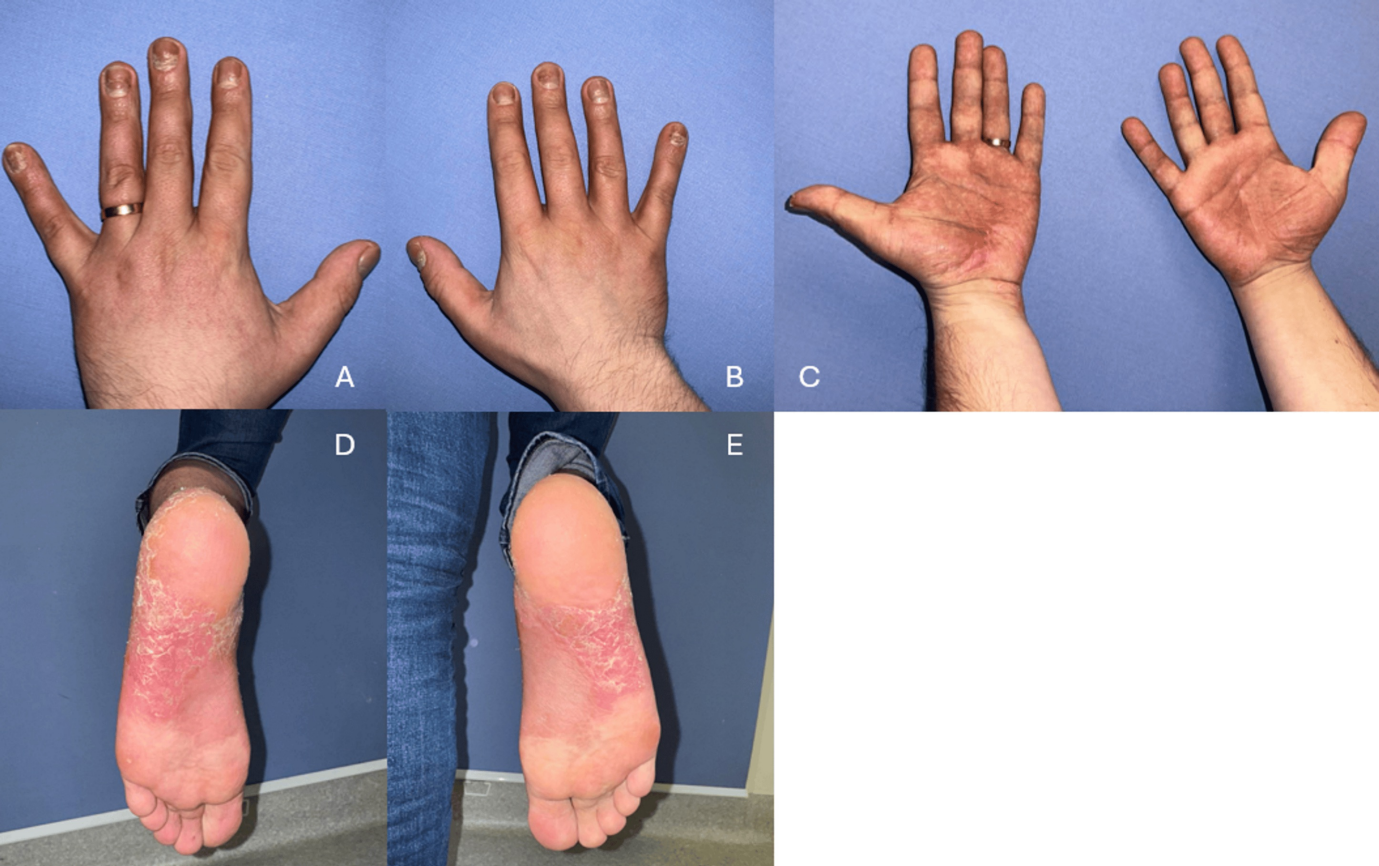
Palmoplantar and nail lesions in October 2025 (A) Dorsal view of the left hand and fingernails; (B) dorsal view of the right hand and fingernails; (C) palmar view of both hands; (D) plantar view of the left foot; (E) plantar view of the right foot.

**Figure 4:**
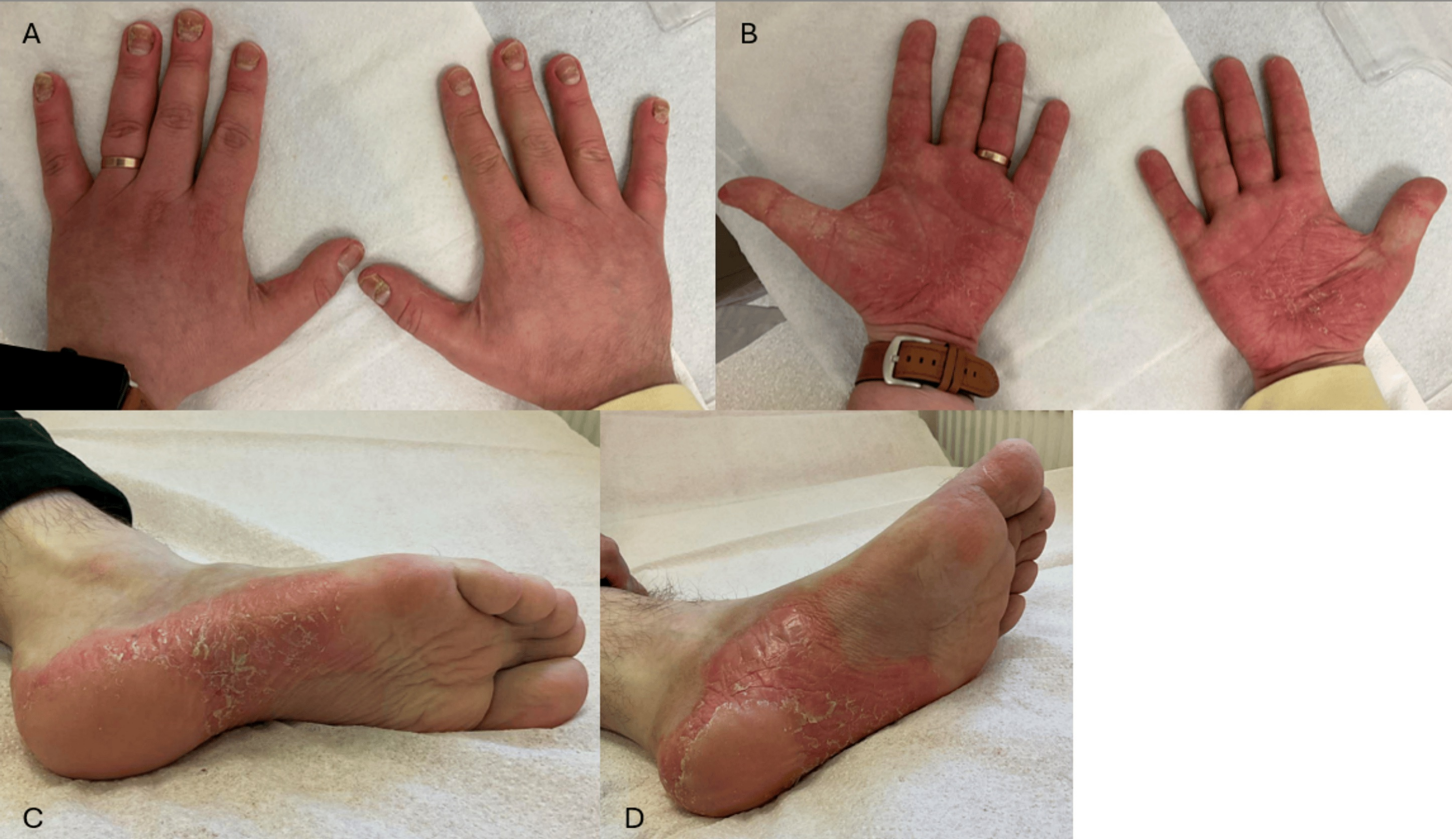
Palmoplantar and nail lesions in November 2025 (A) Dorsal view of both hands and fingernails; (B) palmar view of both hands; (C) plantar view of the right foot; (D) plantar view of the left foot.

This approach yielded a partial and transient response, with palm lesions regressing to mild residual scaling; reduced plantar erythema and scaling despite persistent pustules (Figures 3A-3E, Figures 4A-4D); and ongoing scalp erythema and scaling, followed by relapse one week after completion of phototherapy. Digital dermoscopy continued to show erythema, dotted capillaries, and pustules on the palms and soles, while the scalp demonstrated a few minimally infiltrated erythematosquamous plaques up to 2 cm in diameter. Systemic acitretin was considered but not initiated due to abnormal blood biochemistry (Table 1). Following a multidisciplinary discussion, initiation of the interleukin-17 (IL-17) inhibitor ixekizumab at the standard regimen was recommended, although the patient declined further biologic therapy.

**Table 1:** Biochemical blood abnomalities

Test	Value	Normal range
Gamma-glutamyl transferase	73 IU/L	≤36 IU/L
Total cholesterol	5.84 mmol/L	<5.2 mmol/L
Low-density lipoprotein cholesterol	4.1 mmol/L	<3.0 mmol/L
Non–high-density lipoprotein cholesterol	4.14 mmol/L	<3.4 mmol/L

In addition, recurrent bilaterally draining groin nodules developed in July 2025, with one episode requiring surgical incision and drainage in September, raising suspicion for hidradenitis suppurativa due to lesion morphology and intertriginous location. Antibacterial therapy was administered with cefadroxil, followed by amoxicillin/clavulanic acid and ciprofloxacin after the culture identified *Escherichia coli*. Subsequent recurrences were clinically milder but persisted, and immunological evaluation revealed no evidence of immunodeficiency. Another notable finding was extensive fingernail involvement, affecting most nails and manifesting as brittleness, oil-drop discoloration, pitting, and marked subungual hyperkeratosis. Systemic treatment with terbinafine for the nail disease was not initiated because the patient planned a prolonged stay in a sunnier climate and expressed concern about potential phototoxic adverse effects.

In November 2025, due to recurrent intraretinal fluid in the RE, a repeat intravitreal dexamethasone implant was administered. Dermatologic management was continued with topical treatment, including emollients for body skin, topical GCS, tacrolimus on alternating days, keratolytic shampoos for the scalp, and mometasone furoate solution. Subsequent follow-up was not available, as the patient had relocated abroad.

## Discussion

Diagnostic role of HLA-A29

Birdshot chorioretinopathy is characterized by an almost unique genetic signature, with HLA-A29 identified in more than 90% of affected individuals [2]. Although HLA-A29 is relatively common in populations of European ancestry, with a prevalence of approximately 7% to 9%, birdshot chorioretinopathy is exceedingly rare in the general population, with an estimated annual incidence of 0.035 cases per 100,000 individuals. Accordingly, HLA-A29 positivity alone has limited utility as a screening or sole confirmatory marker and is more likely to reflect genetic susceptibility that requires additional genetic factors and exogenous triggers for clinical expression [4-6]. In one consensus-based diagnostic framework, HLA-A29 positivity is regarded as an essential diagnostic criterion, whereas in other classification systems, it is considered a feature that enhances diagnostic specificity rather than an absolute requirement in every clinically compatible case [2,7-9]. In the Standardization of Uveitis Nomenclature classification criteria, HLA-A29 testing is applied alongside clinical and imaging features because in multifocal choroiditis, the combination of HLA-A29 positivity with characteristic birdshot lesions or supportive indocyanine green angiography (ICG) findings increases classification specificity [2]. Therefore, in the present case, a diagnosis of HLA-A29-negative birdshot chorioretinopathy was retained, as the clinical phenotype was highly consistent with the disease and the principal alternative causes of birdshot-like posterior uveitis had been excluded during the diagnostic work-up, including sarcoidosis and syphilis [1,2].

GCS limitations

In the course of birdshot chorioretinopathy, CME is one of the most common causes of central visual impairment. Systemic GCS is used in routine clinical practice as a short-term measure to rapidly control intense inflammation, yet monotherapy is considered insufficient over time and does not prevent disease relapses [10]. In addition, systemic GCS exerts mineralocorticoid effects that are associated with sodium and fluid retention, and exogenous GCS are recognized as a triggering or exacerbating factor for central serous chorioretinopathy. Consequently, changes in retinal fluid dynamics may reflect a steroid-related predisposition to serous exudation [11,12]. In the presented case, the rise in IOP after intravitreal dexamethasone implantation was clinically relevant, although such pressure elevation is a known and relatively frequent adverse effect of intravitreal GCS therapy [13,14].

TNF-α inhibition paradox

Although TNF-α blockade represents a rational strategy for controlling systemic inflammation in autoimmune diseases, it may paradoxically provoke psoriasiform or psoriatic lesions as an adverse drug-triggered reaction [15,16]. Paradoxical psoriasis may manifest as an exacerbation of pre-existing psoriasis or, more commonly, as *de novo* psoriatic eruptions. Typical phenotypes include palmoplantar pustular lesions and plaque and guttate psoriasis, as well as scalp and nail involvement [15]. In a systematic review, the largest proportion of reported cases was associated with infliximab, followed by adalimumab. The authors proposed that this distribution likely reflects the broader use of infliximab and its earlier introduction into clinical practice compared with adalimumab [17].

The onset of paradoxical psoriasis is often delayed and typically occurs within the first year, approximately 11 months after initiation of a TNF-α inhibitor, a pattern that is consistent with a multifactorial pathogenesis and a role for additional triggers [18]. In the presented patient, palmoplantar pustular disease represents a common variant of paradoxical psoriasis, whereas the biopsy finding of spongiotic dermatitis with epidermal Langerhans cell aggregates is more supportive of allergic contact dermatitis and raises the possibility of a mixed mechanism [15,17,19]. This combination of histopathological features and a pustular palmoplantar course supports patch testing to identify contact allergens and subsequent avoidance, and it may also explain a partial or short-lived response to topical GCS and phototherapy [17,19,20]. Management algorithms for paradoxical psoriasis recommend discontinuation of the responsible TNF-α inhibitor and, when indicated, switching to a different biologic class, because within-class substitution has limited effectiveness and has been associated with lesion recurrence [17,21,22].

In the context of this case, the development of psoriasis is clinically relevant within a broader spectrum of paradoxical adverse events, as TNF-α inhibitors may not only induce psoriasiform lesions but also be linked to other inflammatory manifestations typically treated with these agents, including uveitis and granulomatous reactions [23,24]. This inversion of the expected immune trajectory is most commonly explained by sustained type I interferon signaling in the skin during TNF-α blockade, which is associated with activation of plasmacytoid dendritic cells and excessive interferon-α production, leading to lesions even in the absence of prior psoriasis [3,16,25]. Clinically and histologically, paradoxical psoriasis often resembles conventional psoriasis, yet its pathogenesis is considered heterogeneous and remains debated. Two overlapping mechanistic frameworks are commonly discussed: a hypersensitivity reaction to TNF-α inhibitors linked to anti-drug antibody formation and a genetically conditioned cytokine imbalance in which maturation of conventional and plasmacytoid dendritic cells is altered, interferon-α production increases, and interleukin 23 (IL-23) and T helper 17 (Th17) signaling is amplified. In contrast to classic psoriasis, biopsies may therefore show a broad spectrum from psoriasiform changes to eczematoid, spongiotic patterns, creating a persistent diagnostic grey zone between these mechanisms [21,26-28].

Biologic therapy at the eye-skin interface

When selecting a steroid-sparing strategy, the interleukin 6 (IL-6) inhibitor tocilizumab has been associated with reductions in retinal vasculitis and central retinal thickness in refractory birdshot chorioretinopathy, as assessed by FA and OCT. However, choroidal inflammatory dynamics assessed by ICG appear less favorable, suggesting that the effect of tocilizumab on the choroidal component may be limited [29,30]. From a dermatological perspective, IL-17 pathway blockade is frequently considered for palmoplantar pustulosis, given the presence of neutrophil-rich pustules and transcriptomic evidence of activation of IL-23, interleukin 36 (IL-36), and neutrophil chemotaxis-related chemokines, including CXC chemokine ligands 1, 2, and 8 [31-33]. Although the Th17 and IL-17 axis is regarded as relevant to the immunopathogenesis of noninfectious uveitis and has been investigated as a steroid-sparing systemic approach, its benefit in relapse prevention remains insufficiently defined. Therefore, selecting IL-17 blockade for a dermatological indication should be pragmatically combined with ophthalmologic monitoring and assessment of the risk-benefit balance [34-37]. Selective IL-23p19 inhibitors are considered high-efficacy biologic options in psoriasis vulgaris treatment guidelines, and this drug class is commonly associated with achieving at least a 90% reduction in the Psoriasis Area and Severity Index (PASI) from baseline, reflecting substantial cutaneous improvement. Although targeting the IL-23 axis in noninfectious uveitis is not yet established, pathogenic and biomarker data provide a justification for considering its potential relevance [38-40]. Studies have demonstrated increased IL-23 levels in serum or intraocular fluids during active uveitis, including elevated serum IL-23 in untreated birdshot chorioretinopathy, and, mechanistically, IL-23 sustains Th17 immune responses and amplifies inflammatory cascades [38]. Nevertheless, a case of uveitis progression during treatment with guselkumab (IL-23p19 inhibitor) has been reported, and IL-23p19 inhibition is therefore approached with caution in patients with active or recently active ocular inflammation [41].

Ustekinumab may also be considered a potential steroid-sparing option: unlike selective IL-23p19 inhibitors, ustekinumab binds the p40 subunit shared by interleukin 12 and IL-23, thereby providing a mechanistic basis for modulation of the T helper 1 and T helper 17 inflammatory axis [42,43]. Although evidence in noninfectious uveitis remains limited, ustekinumab may represent a biologically plausible alternative in selected cases, provided that treatment selection is individualized and accompanied by ophthalmologic monitoring [44-46].

## Conclusions

Intravitreal dexamethasone implantation produced an improvement in CME, but its role was limited by marked steroid-associated IOP elevation. Adalimumab provided insufficient CME control and was associated with paradoxical psoriasis, leading to treatment discontinuation. Topical therapy and phototherapy achieved only partial, short-lived benefit, and escalation to an alternative biological therapy class compatible with ocular inflammatory risk was considered but declined by the patient. Overall, this case underscores the need for coordinated ophthalmology-dermatovenereology management, with treatment choices guided by the dominant organ involvement, adverse event risk, and patient preferences.
